# Fear of crime and the environment: systematic review of UK qualitative evidence

**DOI:** 10.1186/1471-2458-13-496

**Published:** 2013-05-24

**Authors:** Theo Lorenc, Mark Petticrew, Margaret Whitehead, David Neary, Stephen Clayton, Kath Wright, Hilary Thomson, Steven Cummins, Amanda Sowden, Adrian Renton

**Affiliations:** 1 Department of Social and Environmental Health Research, London School of Hygiene & Tropical Medicine, London, UK; 2 Department of Public Health and Policy, University of Liverpool, Liverpool, UK; 3 Centre for Reviews and Dissemination, University of York, York, UK; 4 MRC Social and Public Health Sciences Unit, Glasgow, UK; 5 Department of Social and Environmental Health Research, London School of Hygiene & Tropical Medicine, London, UK; 6 Institute for Health and Human Development, University of East London, London, UK

**Keywords:** Crime/psychology, Fear, Review, Environment design, Qualitative research

## Abstract

**Background:**

The fear of crime may have negative consequences for health and wellbeing. It is influenced by factors in the physical and social environment. This study aimed to review and synthesize qualitative evidence from the UK on fear of crime and the environment.

**Methods:**

Eighteen databases were searched, including crime, health and social science databases. Qualitative studies conducted in the UK which presented data on fear of crime and the environment were included. Quality was assessed using Hawker et al.’s framework. Data were synthesized thematically.

**Results:**

A total of 40 studies were included in the review. Several factors in the physical environment are perceived to impact on fear of crime, including visibility and signs of neglect. However, factors in the local social environment appear to be more important as drivers of fear of crime, including social networks and familiarity. Broader social factors appear to be of limited relevance. There is considerable evidence for limitations on physical activity as a result of fear of crime, but less for mental health impacts.

**Conclusions:**

Fear of crime represents a complex set of responses to the environment. It may play a role in mediating environmental impacts on health and wellbeing.

## Background

Most research on crime and health hitherto has focused on the direct health impacts suffered by victims of crime, particularly violent crime [1-3]. However, the indirect effects of crime and its broader harms on individuals and communities may also have important impacts on wellbeing. Fear of crime is of particular interest here, as it has been shown in several studies to have a modest, but consistently significant, association with health and wellbeing outcomes at the individual level, although there is still some controversy about the meaning of this association and the direction of causality underlying it [4-6].

A number of studies have found that fear is only weakly correlated with objective measures of crime, suggesting that fear of crime is not simply a response to high crime rates [7,8]. Fear appears to be more consistently associated with conditions in the physical environment, particularly signs of neglect such as litter and graffiti [4]; it has also been hypothesized to correlate with social factors such as social cohesion, although the findings here are more equivocal.

These findings suggest, first, that fear of crime may have an impact on health and wellbeing at a population level, independently of the direct impacts on crime on victims; and, second, that fear of crime is at least in part a response to factors in the social and physical environment. Therefore, fear of crime may be of interest to researchers in public health as a potential pathway mediating the effect of community-level environmental factors on health and wellbeing.

Qualitative research may of value in understanding the place of fear in individuals’ lives, and the determinants which shape it. From a public health perspective, qualitative research may also help to fill in the gaps in our understanding of how fear of crime influences wellbeing outcomes, and to gain a greater insight into how both relate to environmental factors. This can help to illuminate one area of the complex pathways through which environmental determinants impact on health and wellbeing outcomes.

### Aim

The aim of this review was to synthesize qualitative evidence from the UK on fear of crime and the environment. The review focuses on UK evidence because qualitative research from other countries may be of limited applicability, and because a substantial body of good-quality qualitative evidence from the UK exists.

## Methods

### Searching

We searched the following databases. Searches were conducted between November 2010 and January 2011. All sources were searched from inception to the most current records.

•ASSIA

•CINAHL

•Conference Proceedings Citation Index

•Criminal Justice Abstracts

•Dissertation Abstracts

•EconLit

•Embase

•ERIC

•HMIC

•Inside Conferences

•Medline

•NCJRS

•PsycInfo

•Science Citation Index

•Social Policy & Practice

•Social Science Citation Index

•Sociological Abstracts

•Urban Studies Abstracts

The search strategy used took the following form:

((fear of crime) OR (crime) OR (antisocial behaviour)) AND ((built environment) OR (built environment interventions))

The full Medline search strategy can be found in web-only Additional file 1. Searches for other databases used a modified form of the Medline search strategy. No further limitations (e.g. by language or date of publication) were used in the searches.

The following additional sources were also used to locate studies:

•Google and Google Scholar (using a simplified version of the main search string and screening the first 50 hits from each);

•citation chasing from the studies included in the review;

•citation chasing from relevant systematic reviews located by the searches (i.e. which met all the inclusion criteria except that relating to study design);

•searches of websites of government bodies, research groups and other relevant organisations; and

•consultation with members of the research team and the Advisory Group.

### Screening

Two reviewers coded an initial sample of records independently, with differences resolved by discussion and reference to a third reviewer where necessary. In total, 10% of the records were screened by two reviewers independently. The remaining abstracts were screened by one reviewer alone.

The following inclusion criteria were applied:

(1) Does the study report substantive data on the fear of crime?

(2) Does the study report substantive data on some aspect of the built environment?

(3) Is the study a primary qualitative study e.g. interviews, focus groups, ethnography?

(4) Was the study conducted in the UK?

The full text of all studies which met the inclusion criteria at abstract stage was retrieved and re-screened using the same criteria. Of the full text studies, 50% were screened independently by two reviewers, with differences resolved by discussion; the remainder were screened by one reviewer alone.

### Data extraction and quality assessment

Data were extracted from the published study reports using a standardized form which included information on the context and setting of the study, the population, the methodology and the findings. Findings data were extracted only for direct quotes from participants cited in the study reports. Data extraction and quality assessment for all studies were carried out by a single reviewer and a sample double-checked in detail by a second reviewer.

Quality assessment for the qualitative review was carried out using Hawker et al.’s framework [9]. This tool allows for a systematic assessment by the reviewer for standard of reporting as well as appropriateness of methods. The tool includes an assessment of nine domains: abstract and title; introduction and aims; method and data; sampling; data analysis; ethics and bias; results; transferability/generalizability; implications; and usefulness. Each domain was scored from 1 (very poor) to 4 (good), giving an overall score between 9 and 36. Overall quality ratings were then assigned as follows: high quality (A), 30–36 points; medium quality (B), 24–29 points; low quality (C), 9–24 points. Studies were not excluded or given less weight in the synthesis on the basis of the quality assessment scores.

### Data synthesis

The quotes reported in the identified publications were coded thematically using a broad coding framework. Although, as described above, the initial aim of the review was to focus on perceptions of the physical built environment, it became clear at an early stage in the analysis that the social environment would also need to be included to provide a coherent synthesis. Thus, the framework included the following categories: determinants of fear in the physical environment; determinants of fear in the social environment; and consequences of fear. A grounded theory approach was used to allow for the emergence of new themes or codes within the initial coding framework.

## Results

**Figure 1:**
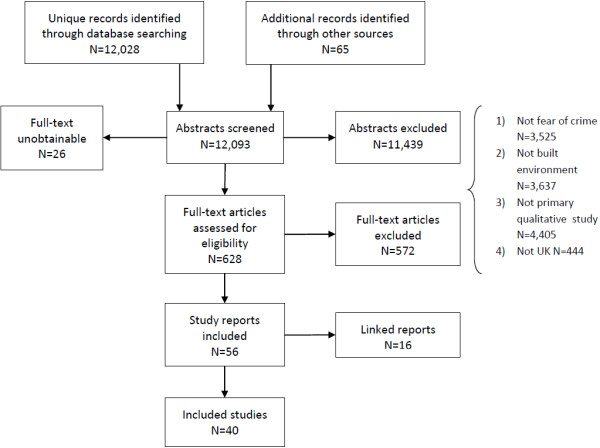
Flow of literature through the review.

The flow of literature is presented in Figure 1. Forty studies were included in the review. Table 1 shows the studies, where they were conducted, the primary research question or focus, the data collection methods used, the population and the quality rating assigned.

**Table 1:** Characteristics of the studies (N=40)

**First author and reference**	**Location**	Data collection methods*	**Research question or focus**	**Population included**	**QA**
Airey [10,11]	Edinburgh	Individual interviews	Effects of place on wellbeing, esp. physical incivilities	Women aged 45-59	A
Alexander [12,13]	Newcastle-upon-Tyne	Focus groups and participatory methods	Effects of FoC on social inclusion and citizenship	Young people aged 16-25	C
Bannister [14]	Glasgow	Focus groups	Relations between physical environment and FoC	General population	C
Burgess [15-17]	Hertfordshire; nr Nottingham	Focus groups and participant observation	Perceptions of woodland and associated FoC	General population, esp. women	B
Cozens [18]	S Wales	Questionnaires, focus groups, virtual reality ‘walk-through’	Perceptions of safety in railway stations	General population	C
Crime Concern [19]	NR	Focus groups	Perceptions of safety in pedestrian journeys	General population	C
Crime Concern [20]	Various (England & Wales)	Focus groups, escorted journeys	Perceptions of safety and FoC on public transport	General population	C
Davis [21]	Birmingham	Questionnaires, focus groups	Perceptions of risk, esp. relating to transport	Children and young people aged 9-14	C
Day [22]	Glasgow and environs	Individual interviews, focus groups, observation	Effects of physical environment on wellbeing	Older people aged >60	A
Dixey [23]	Leeds	Individual interviews	Parents’ perceptions of child safety	Mothers of primary-school-aged children	B
Farrall [24-27]	London; Glasgow	Individual interviews	Perceptions of crime and the environment	General population	B
Girling [28-30]	Macclesfield; Prestbury (Cheshire)	Individual interviews, focus groups, observation	Perceptions of crime	General population	B
Goodey [31,32]	N England	Questionnaires, focus groups	Gender differences in FoC	Young people aged 11-16	C
Hollway [33,34]	NR	Individual interviews	Experiences of FoC	General population	C
Hopkins [35]	Glasgow	Individual interviews, focus groups	Experiences of FoC	Young Muslim men aged 16-25	C
Innes [36]	Blackpool; Oldham; London	Individual interviews	Perceptions of crime, anti-social behaviour and physical incivilities	General population	C
Jones [37]	NR	Focus groups	Perceptions of risk and constraints on behaviour; ethnic differences	Young women aged 11–14, most Asian	B
Koskela [38-40]	Edinburgh	Individual interviews	Relation between FoC and built environment	Women	B
Little [41]	Devon	Individual interviews	FoC in rural areas	Women	B
Mitchell [42]	NE England	Individual interviews	Mothers’ perceptions of risk for children	Young mothers aged 15-24	C
Moran [43-45]	Manchester; Lancaster	Individual interviews, focus groups	Fear of violence and its relation to spatiality	Lesbians and gay men	C
Nayak [46]	NE England	Questionnaires	Experiences of FoC	Young people aged 12-15	C
Nelson [47]	Cardiff; Gloucester; Worcester	Individual interviews	Perceptions of security shutters	General population	C
Pain [48,49]	Newcastle-upon-Tyne and environs	Individual and couple interviews	Perceptions of crime	Older people	C
Pain [50]	Newcastle-upon-Tyne	Focus groups	Perceptions of safety	General population	B
Pain [51,52]	Gateshead	Focus groups, questionnaires, participatory methods	Perceptions of risk and leisure time; role of mobile phones	Young people aged 10-16	C
Pain [53]	Northumberland	Focus groups, observation	Perceptions of street lighting and FoC	General population	B
Parry [54]	W Midlands	Focus groups	Effects of community factors on health	Young people aged 16–20 and older people aged >60	B
Seabrook [55]	N England	Pair interviews, participatory methods	Perceptions of risk, place and leisure time	Girls and young women aged 10–17	C
Squires [56]	Brighton	Individual interviews	Evaluation of CCTV system	General population	C
Taylor [57]	Manchester; Sheffield	Focus groups	Wellbeing and social change	General population	C
Trayers [58]	SW England	Focus groups	Views on planned neighbourhood renewal intervention	General population	A
Turner [59]	Glasgow and environs	Individual interviews, focus groups	Perceptions of risk and safety	Children and young people aged 8–14	A
Valentine [60-63]	Reading	Individual interviews, focus groups	Fear of male violence and perceptions of public space	Women	A
Valentine [64]	Peak District	Individual and couple interviews	Parents’ views of children’s safety in rural area	Parents of 8-11-year-old children	C
Walklate [65,66]	Salford	Individual interviews, focus groups, observation, content analysis	Perceptions of risk, FoC and community	General population	C
Waters [67]	Glamorgan; Loughborough	Questionnaires, focus groups, virtual reality ‘walk-throughs’	Perceptions of safety on university campuses	University staff and students	A
Waters [68,69]	S Wales	Focus groups, virtual reality ‘walk-throughs’	Perceptions of crime, FoC and community	Older people aged >65	A
Watson [70]	Leeds	Individual interviews, observation	Experiences of risk w/r/t leisure time	Young mothers	C
Whitley [71,72]	London	Individual interviews, focus groups, observation	Impact of FoC on mental health	General population; people with mental health problems	A

*For mixed-methods studies, this column refers to the qualitative component only. Abbreviations: CCTV = closed-circuit television; FoC = fear of crime.

### Determinants of fear in the physical environment

Several factors in the physical environment are described by participants as relevant to the fear of crime. Some participants see physical security measures, such as locks, fencing or secure entry systems, as reducing fear [27,71]. However, measures in public space such as shutters and security gates are often seen as increasing fear, and as creating an unpleasant and hostile atmosphere more generally [28,47,57,67]. Excessive security measures in the home are also seen as unwelcoming and depressing, with several participants using the metaphor of a fortress or prison [26,28,39,67,71]. In particular, several participants express a sense of anger at the need for such security measures [24,26,39].

Street lighting is also frequently discussed in the studies. Many participants report feeling more fearful in poorly lit locations [19,20,28,38,40,50,53,60],[67] and at night [27,28,36,41,47,57]. Lighting appears to be relevant to fear in two ways. First, it increases visibility and is thought to reduce potential hiding places for attackers [38,60,67]. Second, it gives a more pleasant and welcoming impression of the environment, partly by acting as an indicator of the presence of other people [20,67]. In some cases, participants report that the effects of lighting are outweighed by other factors which impact on fear: “I mean when I was a child we lived in the country and it was all dark lanes with no lights, but we never felt afraid” [60]. Participants in two studies express scepticism about the effectiveness of lighting as a fear reduction strategy [38,53]. Finally, specific aspects of lighting such as colour and brightness may also make be relevant to fear [19,50,53,67].

Lighting also relates to the sense that the environment allows visibility (what criminologists call ‘natural surveillance’). Places which are not visible because they are isolated [18,20,38], or sight-lines which are obstructed by vegetation, landscaping or poorly designed buildings are perceived to increase the risk of attack, and hence fear [19,20,38,67]. Such obstructions to visibility also create a feeling of being ‘trapped’; by contrast, a sense of ‘openness’ in the environment is reassuring [15,67].

Closed-circuit television (CCTV) is relatively rarely mentioned in the studies. A few participants express support for CCTV in general terms [18,20,28,50], but few say that it reduces fear, and several are sceptical about its effectiveness in reducing crime [20,47,50,56]. Several participants in Squires’ evaluation of CCTV are strongly critical of it, seeing it as an inadequate substitute for more substantive measures to reduce crime [56].

Dirt, decay, graffiti, litter and other signs of neglect of the environment (what criminologists call ‘physical incivilities’) are widely seen as drivers of fear [18-20,24,31,36,41,54,60,67],[69], for several reasons. Problems in the physical environment are seen to indicate a lack of commitment to social norms [24,67,69]. Problems such as graffiti or litter are associated with environmental indicators of socio-economic disadvantage (such as high-rise housing) as part of a more general sense of ‘rough’ areas [24,60,67]. More broadly, a pleasant physical environment is thought to contribute to an overall sense of wellbeing, and thus safety [24,54,71].

Finally, places where few other people are around, either because of the time of day or because of patterns of land use, are widely experienced as fear-inducing [15,19,20,38,40,50,67].

### Determinants of fear in the social environment

An important factor relevant to fear is the extent to which one is familiar with an area. Many participants report feeling less fearful in their own area, or areas they know well, than elsewhere. “How true it is that one often feels safer in your local area… I just feel safer because it’s my local area and I know what happens there and I feel more confident” [19]. Participants describe factors which may increase fear for outsiders but are not seen as threatening by insiders. “I think it’s all right round here, I mean you see gangs of kids but they’re only young and it doesn’t bother me because it’s familiar, I mean I’ve always lived round here” [70]. In Bannister’s study, participants were asked to mark areas seen as unsafe on a map; most saw their own areas as safe and areas not known well as unsafe, such that a large proportion of residential areas were seen as unsafe by at least one participant, but none by all the participants [14].

Much of the protective effect of familiarity has to do with having strong social networks locally. Many participants report that they do not feel fearful in their own area because they know many people and are long-term residents in the area [26,27,36,48,60]. “Everyone knows everyone, so you’re not a stranger in your own town. And you just feel so safe, just in your own street and your own area” [36]. Conversely, strangers who come to the area from elsewhere are often the object of fear [27,28,60,64].

Young people, especially when ‘hanging about’ in groups, are often perceived as threatening [19,20,28,41,46,56,64], as are people using alcohol or drugs [19,20,22,24,36,50,60,69], especially large groups at pub closing time [28,45,50,60].

There is some evidence suggesting differences in fear between women and men. Women tend to express greater fear, and the focus of their fear is virtually always men [19,60]. To some extent this reflects differences in the crimes feared, with rape or sexual assault being the focus of fear for women [31,40,60], and perceived differences in physical vulnerability [48,60]. It may also be affected by ‘vicarious’ fear expressed by husbands or boyfriends or parents, which may lead to restrictions on women’s activities [21,27,31,37,48,60].

However, it also seems to reflect women’s experience of everyday harassment and relatively minor crimes such as indecent exposure [15,60]. Women’s fear appears to be more pervasive and inescapable than men’s, with several women participants expressing doubt that fear can ever be meaningfully addressed [40,60]. “You’re never safe at any time. If somebody wants to go out and attack a woman, they’ll do it” [40]. These points have parallels in the experience of ethnic minority participants [35] and lesbian and gay participants [43,45], several of whom also express a pervasive fear which is partly driven by the everyday experience of discrimination and harassment.

### Consequences of fear

Relatively few participants see fear as having serious mental health impacts, although several report some degree of psychological stress as a result of fear [10,57,60]. Those who do report serious mental health consequences tend to be victims of serious violent crimes, particularly sexual or hate crimes [19,56,60], or people with pre-existing mental health problems [71]. Some participants also see fear as impacting on health as part of a broader nexus of disadvantage [10].

A much more widely perceived consequence of fear is to limit people’s activities, including social and cultural activities, sometimes leading to social isolation [12,20,50,54,55,57,71]. Participants from across the population report such limitations, but they appear to be more serious for women, older people and people with disabilities. Of particular concern from a health perspective are limitations on outdoor physical activity, especially walking and cycling, which are reported by several female participants [15,39,40,60]. Parents also report placing serious restrictions on children’s activities [21,23,37,39,60,64], even though both parents [23,64] and children [21,37] recognize the problematic effects of such restrictions on children’s independent mobility. Again, parental restrictions are often more serious for girls and young women [21,31,37,60].

More broadly, fear of crime is seen to contribute to the process by which disadvantaged areas gain a reputation as dangerous or ‘rough’, which can contribute to the social stigmatization of residents of those areas [10,12,24,56,73].

## Discussion and conclusion

This is the first review to draw together the large body of UK qualitative evidence on fear of crime and the environment. Although this review is exploratory in nature and does not support strong conclusions, it helps to fill out the available theories and quantitative data which suggest that fear of crime is associated with poorer health outcomes, and that it may mediate determinants of health and wellbeing in the physical and social environment [4]. The findings of this review suggest some plausible pathways through which a number of factors in the physical and social environment may have an impact on fear, and in turn may influence wellbeing, particularly through restrictions on activities. Moreover, the findings suggest that fear of crime may play a role in generating health inequalities, since certain groups appear to be more seriously affected by fear; gender is the most obviously relevant dimension here, although age, ethnicity, sexuality and disability may all also play a role.

The relations between environmental factors and fear are complex. Aspects of the physical built environment are clearly relevant to fear to some extent, but fear often relates more directly to the environment’s social meanings than to its physical form. For example, familiarity with and social inclusion in a given context may largely nullify the potentially fear-inducing physical features of that context. Conversely, physical factors such as litter and graffiti increase fear mainly because they are taken to indicate low social cohesion and/or socio-economic disadvantage. (This applies particularly to residential areas; in public areas, such as shopping streets, parks, or public transport, the role of the physical environment may be greater). Nonetheless, it appears that most of the social factors which are relevant to fear of crime are spatially localised. This social mediation of physical cues also means that different people, or population subgroups, may come to different conclusions about the same physical environmental factors.

The social drivers of fear are complex and often contested. Several themes in the data, such as the fear of young people ‘hanging about', appear to represent a conflict between different group norms about the use of public space. Moreover, the findings on inequalities, particularly by gender, lend some support to theories of ‘spirit injury’ which posit an important role for systemic discrimination in the genesis of fear of crime [74,75]. Such theories suggest that the latent structural violence involved in maintaining social inequalities may be as important as the manifest violence measured in crime statistics in understanding fear and its impacts on wellbeing.

Although this review was carried out according to full systematic review methodology, it has some limitations. Only a thematic analysis, focusing on directly reported primary data, was carried out. The search terms and inclusion criteria, which focused on fear of crime and the physical built environment, may have excluded a number of relevant studies (e.g. studies which focused on the social environment alone). The primary studies are heterogeneous in many respects, and generalizations across them may have limited validity.

These limitations aside, this review suggests that fear of crime may have some role to play in mediating the impact of physical environmental factors on wellbeing, particularly by acting as a barrier to outdoor physical activity. However, the ways in which the environment influences fear appear to be be complex. The findings suggest that physical environmental change alone, and interventions which focus narrowly on crime reduction, are likely to have limited success in addressing fear and its effects on wellbeing. Approaches which engage with the broader social contexts of fear of crime – including socio-economic disadvantage and its symbolic meanings, and inequalities with respect to gender and ethnicity – appear to be more promising.

## Competing interests

No authors have any competing interests.

## Authors’ contributions

MP and MW conceived the study, with input from HT, SCu, AS and AR. KW ran the literature searches. Screening and data extraction were carried out by TL, SCl and DN. Data analysis was carried out by TL. All authors contributed to the interpretation of the findings and approved the final report.

## Pre-publication history

The pre-publication history for this paper can be accessed here:

http://www.biomedcentral.com/1471-2458/13/496/prepub

## Supplementary Material

Additional file 1 MEDLINE search strategy. Click here for file
